# Genetically Predicted Immune Cell Traits Mediate the Causal Association Between Plasma Metabolites and Colorectal Cancer

**DOI:** 10.7150/jca.101011

**Published:** 2025-01-01

**Authors:** Liye Zhu, Qiting Ning, Jiang Xue, Shanpei Huang, Xingmei Chen, Xinze Qiu, Ni Chen, Shengmei Liang, Jiean Huang, Shiquan Liu

**Affiliations:** 1 Department of Gastroenterology, The Second Affiliated Hospital of Guangxi Medical University, Nanning, Guangxi 530007, China.; 2 Spinal and Orthopaedic Surgery Ward, The First Affiliated Hospital of Guangxi Medical University, Nanning, Guangxi 530021, China.

**Keywords:** Plasma metabolites, Immune cell traits, Colorectal cancer, Mendelian randomization, Mediation analysis, Single-cell RNA sequencing

## Abstract

**Background:** Relevant studies have demonstrated that plasma metabolites and immune cell characteristics are closely related to colorectal cancer (CRC). However, the causal relationship among these factors remains unclear, particularly regarding whether immune cell traits mediate the causal link between plasma metabolites and CRC.

**Methods:** This study employed a two-step, two-sample Mendelian randomization (MR) using summary data from genome-wide association studies (GWAS) to assess causal associations between 1,400 plasma metabolites, 731 immune cell traits, and CRC. Additionally, it evaluated the mediating effect of immune cell traits and utilized single-cell RNA sequencing (scRNA-seq) to analyze immune cells infiltration in CRC, assess their metabolic functional changes and their interactions with CRC cells.

**Results:** Univariable two-sample MR analysis revealed causal relationships between 49 plasma metabolites and CRC, as well as between 36 immune cell traits and CRC. Two-step MR analysis revealed that two plasma metabolites (Sphingomyelin (d18:1/22:1, d18:2/22:0, d16:1/24:1) and 16α-hydroxy-DHEA-3-sulfate) influence CRC through two immune cell traits (SSC-A on CD14+ monocyte and CD3 on CD28- CD8+ T cell). Among these, SSC-A on CD14+ monocyte exhibited the highest mediating effect proportion, at 11.723%. scRNA-seq analysis further confirmed the increased infiltration of CD28- CD8+ T cells and CD14+ monocytes in CRC, along with upregulated sphingolipid metabolism and steroid biosynthesis. These cells were also found to interact with CRC cells, contributing to tumor initiation and progression.

**Conclusion:** This study provides new evidence for the causal relationship between plasma metabolites and CRC, and it identifies immune factors with potential mediating roles. These findings offer new insights for further exploration of the mechanisms underlying the development of CRC.

## Introduction

Colorectal cancer (CRC) is the third most commonly diagnosed cancer worldwide, with a cancer-related mortality rate of 9.4%, placing it second in cancer mortality rates 1. Approximately 20% of CRC patients present with distant metastasis at diagnosis, and the prognosis for metastatic CRC patients remains poor 2. Therefore, research into early intervention and preventative management strategies for CRC is essential to reduce the public health burden. Previous studies have identified obesity, BMI, and total cholesterol as common risk factors for CRC 3, 4. Additionally, metabolites and immune cells are closely associated with the development and progression of CRC 5, 6.

Metabolites are chemical substances produced through metabolic processes within organisms. These substances, including amino acids, carbohydrates, lipids, and so on, are influenced by various factors such as genetics, diet, lifestyle, gut microbiota, and disease. Concurrently, these metabolites are also risk factors for disease and serve as targets for therapeutic interventions 7. Metabolic reprogramming is now recognized as one of the ten hallmark characteristics of tumors 8. As key molecules in the metabolic reprogramming process, metabolites play a significant role in the occurrence and development of tumors. Numerous circulating metabolites have been reported to aid in cancer diagnosis 9 and contribute to tumor development 5. For instance, higher intake of trans fatty acids and a higher n-6/n-3 polyunsaturated fatty acid ratio are associated with an increased risk of CRC 10. Additionally, the inhibition of sphingolipid production can suppress CRC proliferation 11. Serine reverses the inhibitory effects of ADH1C overexpression on CRC, promoting tumor progression 12. Bioinformatics analysis and case-control studies have been conducted to explore the relationship between adiponectin receptor ADIPOR1 single nucleotide polymorphisms (SNPs) and lipid profile parameters in CRC patients. The results indicate that ADIPOR1 rs7539542 is associated with reduced levels of HDL-C, LDL-C, and TC in CRC 13. Additionally, Mendelian randomization (MR) studies identified 1-(1-enyl-palmitoyl)-2-arachidonoyl-GPE (P-16:0/20:4) and arachidonate (20:4n6) as risk factors for CRC 14. Although these studies have highlighted the role of certain metabolites in CRC, further research is needed to elucidate new areas of investigation. Furthermore, there is evidence indicating that metabolites may exert an influence on the functionality of immune cells, thereby promoting immune evasion in tumors 15, 16.

Immune cells, as an important component of the tumor immune microenvironment, interact with surrounding cells and are essential for the spread of CRC. A MR analysis examined the effects of five circulating white blood cell types on CRC risk, revealing that increased eosinophil counts may offer a protective effect against CRC 17. Similarly, Elevated levels of CD8+ T cells, Th1 cells, and NK cells in the tumor microenvironment have been associated with anti-tumor effects and improved prognosis in CRC 6. By releasing granzyme and perforin, CD8+ T cells specifically kill target cells, such as tumor cells 18. Inhibition of sphingomyelin synthase 2, resulting in reduced sphingomyelin production, has been found to inhibit M2-type macrophage polarization and improve CD8+ T cell infiltration, thereby suppressing tumor metastasis 19. However, the role of immune cells in mediating the effects of plasma metabolites on CRC progression remains largely unexplored. In recent years, the swift advancements in single-cell RNA sequencing (scRNA-seq) technology have enabled researchers to meticulously dissect the characteristics of diverse cellular subpopulations during the progression of diseases. scRNA-seq provides a high-resolution map of immune cells, facilitating the precise identification of specific cell populations and their corresponding gene expression signatures 20. Therefore, by harmoniously integrating two-step, two-sample mediation MR with scRNA-seq analysis, this study further elucidates the intricate causal relationships between plasma metabolites, immune cell traits and CRC, thereby deepening our understanding of the pivotal roles these factors play in CRC pathogenesis.

## Material and Methods

### Study design

**Figure 1:**
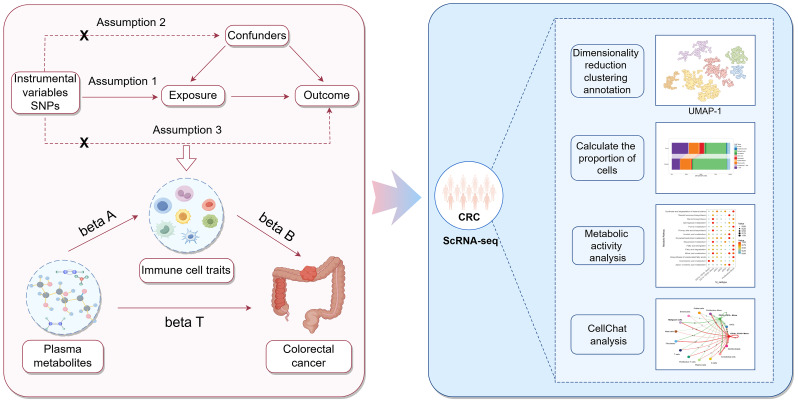
Flowchart of the study analysis.

This study was divided into two main components. The first component utilized a two-step, two-sample MR approach to determine the causal relationship between plasma metabolites and CRC, and to assess whether immune cell traits are mediating this link. In the first step, two-sample MR was employed to evaluate the causal relationships among plasma metabolites, immune cell traits, and CRC, identifying plasma metabolites and immune cell traits that are highly associated with the risk of CRC. The second step was to investigate the casual relationships between the screened plasma metabolites and the screened immune cell traits. Significant associations between plasma metabolites and immune cell traits were identified, and the mediating effect of each immune cell trait on the causal relationship between plasma metabolites and CRC was calculated. The second component involved obtaining scRNA-seq data of CRC tissues and adjacent normal tissues from the GEO database. Following quality control, dimensionality reduction, clustering, and annotation, functional changes in different cell types and subpopulations under tumor conditions were identified and analyzed. Furthermore, the cellular communication and metabolic functional alterations of immune cell subpopulations were explored to validate the roles of specific immune cell subtypes in CRC. The study design is illustrated in Figure 1.

### Source of genome-wide association study data

CRC data were obtained from the FinnGen database (https://finngen.gitbook.io/documentation/v/r10), Data ID: finngen_R10_C3_COLORECTAL_EXALLC. All CRC patients were diagnosed according to ICD-9 or ICD-10 criteria, and individuals in the control group had no history of any other tumors. The database was adjusted for covariates including sex, age, 10 principal components (PCs), Finngen chip version 1 or 2, and legacy genotyping batch 21. A total of 6,847 CRC cases and 314,193 controls were included in the study. Data on 1,400 plasma metabolites were sourced from the GWAS Catalog database (https://www.ebi.ac.uk/gwas/), with identifiers ranging from GCST90199621 to GCST90201020. This dataset comprises 1,091 plasma metabolites and 309 metabolite ratios, including 850 known metabolites categorized into eight classes: lipids, amino acids, xenobiotics, nucleotides, cofactors and vitamins, carbohydrates, peptides, and energy. The remaining 241 are classified as unknown or partially characterized molecules 7. Data for 731 immune cell traits (Ebi-a-GCST0001391 to Ebi-a-GCST0002121) were retrieved from the IEU OpenGWAS project database (https://gwas.mrcieu.ac.uk/). These traits encompass seven cell panels: B cells, dendritic cells (DCs), mature T cells, monocytes, myeloid cell, TBNK, and regulatory T cells (Tregs). Additionally, the 731 immune cell traits include absolute cell counts (AC), relative cell counts (RC), median fluorescence intensity (MFI) reflecting surface antigen levels, and morphological parameters (MP) 22. All samples were from Western populations, selected for inclusion due to their similar genetic structures related to CRC risk.

### Selection of genetic instrumental variables (IVs)

According to the principle of MR, three basic assumptions must be observed when selecting IVs: (1) Relevance: Genetic variants should be significantly associated with the exposure and SNPs should be independent; (2) Exclusion: Genetic variants should not be directly associated with the outcome, influencing it only through the exposure; (3) Independence: Genetic variants should not be related to any known or unknown confounders, which may include environmental, socioeconomic, and other biological factors.

To select appropriate IVs, the following criteria were applied: (1) SNPs significantly associated with genome-wide patterns (significance threshold P < 5×10⁻⁸) were chosen as candidate IVs for CRC. For SNPs related to plasma metabolites and immune cell traits, a significance threshold of P < 1×10⁻^5^ was used; (2) Using the European 1000 Genomes Project as a reference panel, SNPs with linkage disequilibrium (LD) were eliminated (r² < 0.001, clustering window size kb=10000); (3) SNPs significantly associated with the outcome (P < 5×10⁻⁸) were excluded; (4) Palindromic SNPs were removed to ensure that the effects of SNPs on the exposure and outcome correspond to the same allele; (5) The F-statistic was calculated to assess the strength of the IVs, retaining only strong IVs with an F-value greater than 10 (F > 10) and a minor allele frequency (MAF) greater than 0.01 (MAF > 0.01); (6) When original SNP data were missing or of low quality, SNPs with highly similar genetic information (r² > 0.8) were identified using the LDlink tool as substitutes, provided they met the fundamental assumptions of MR analysis.

### Univariate two-sample MR analysis

To evaluate the causal relationships between plasma metabolites and CRC, immune cell traits and CRC, and plasma metabolites and immune cell traits, five MR analysis methods were employed: Inverse Variance Weighted (IVW), Weighted Median, MR-Egger regression, Weighted Mode, and Sample Mode. IVW was used as the primary method, and results with P < 0.05 were considered to have preliminary causal associations. To identify exposure factors with strong causal associations, stricter criteria were applied: (1) IVW-MR method with P < 0.05; (2) consistent direction of OR values across all five MR methods; (3) MR-PRESSO test confirming no outlier interference, with a global P > 0.05; (4) pleiotropy test P > 0.05, indicating no horizontal pleiotropy.

### Mediation MR analysis

In previous analyses, plasma metabolites and immune cells traits with strong causal associations with CRC were identified using two-sample MR, and the total effect (beta T) from plasma metabolite to CRC was calculated. Next, a two-step mediation analysis was conducted to explore immune cells traits that may be mediators of the causal effect of plasma metabolites on CRC. Firstly, using the previously identified plasma metabolites and immune cells traits, we conducted further screening of plasma metabolites that showed strong causal associations with immune cell traits through two-sample MR, calculating their effect values (beta A). Secondly, using the immune cells identified in the first step as exposures, the effect values of immune cell traits on CRC (beta B) were calculated, adjusting for the influence of plasma metabolites by removing the IVs used in the plasma metabolites-immune cell traits MR analysis. Thirdly, a reverse MR analysis was employed to exclude the reverse causal effect of CRC on plasma metabolites. Finally, the mediation effect (beta A × beta B) and its proportion (beta A × beta B / beta T) in the plasma metabolites-CRC pathway were calculated using the "product of coefficients" method. Statistically significant is a P value < 0.05.

### Sensitivity analysis

Sensitivity analyses included tests for pleiotropy and heterogeneity. Pleiotropy was assessed using MR-PRESSO, the Egger regression model and the leave-one-out method. Heterogeneity was assessed by calculating the heterogeneity statistic (Cochran's Q) using the IVW method to assess the variability between the effects of different IVs. Significant heterogeneity or pleiotropy was indicated by a P value less than 0.05.

### Single-cell RNA sequencing analysis

The GSE166555 scRNA-seq dataset was downloaded from the Gene Expression Omnibus (GEO) database (https://www.ncbi.nlm.nih.gov/geo/). This cohort includes tumor and paired normal tissues from 12 CRC patients. High-throughput sequencing for these samples was performed using the 10x Genomics platform and Illumina NextSeq 500 sequencer. The sequencing matrix data were merged using the R package "Seurat V4.4.0," with cell filtering criteria set to exclude cells expressing fewer than 200 or more than 5000 genes, as well as cells with > 5% mitochondrial genes and > 55% ribosomal genes. Subsequently, the data were normalized and standardized, and doublets were removed using the R package "scDblFinder V1.18.0" (with a doublet capture threshold of 0.8% per 1000 cells). The top 2000 most variable genes were selected for dimensionality reduction via PCA, followed by batch correction using harmony, with clustering based on the first 10 principal components. Clustering results were visualized using Uniform Manifold Approximation and Projection (UMAP), and cell clusters were annotated based on marker genes. Cell proportions were calculated with the "scRNAtools v0.1.0" package to evaluate differences between cell types. T cells and monocytes were extracted based on annotation results for further subpopulation analysis, including additional dimensionality reduction, clustering, and annotation of subpopulations. The percentage of each subpopulation within the total cell population was also calculated. Cell-cell communication within CRC subpopulations was analyzed using CellChat (V1.6.1), and metabolic scoring and pathway enrichment of subpopulations were performed using scMetabolism (V0.2.1).

### Statistical analysis

All statistical analyses and bioinformatics processing were conducted using R version 4.4.0, with RStudio as the primary working environment. Relevant R packages were used for data visualization. Differential expression analysis of scRNA-seq data was performed using the Wilcoxon test. Statistically, results with a P-value of less than 0.05 were considered significant.

## Results

### Genetic instrumental variables for exposure

Based on the criteria for selecting genetic instrumental variables, we identified 34,930 significant SNPs (P < 1×10⁻^5^) for 1,400 plasma metabolites, 19,045 significant SNPs (P < 1×10⁻^5^) for 731 immune cell traits, and 12 significant SNPs (P < 5×10⁻^8^) for CRC. Ultimately, 33,854 IVs were chosen for the plasma metabolites-CRC MR analysis, 18,201 IVs for the immune cell traits-CRC MR analysis, and 12 IVs for the CRC- plasma metabolites MR analysis. All selected IVs satisfied the basic assumptions of MR analysis and were strong instrumental variables (Table S1).

### Causal associations of plasma metabolites with CRC

**Figure 2:**
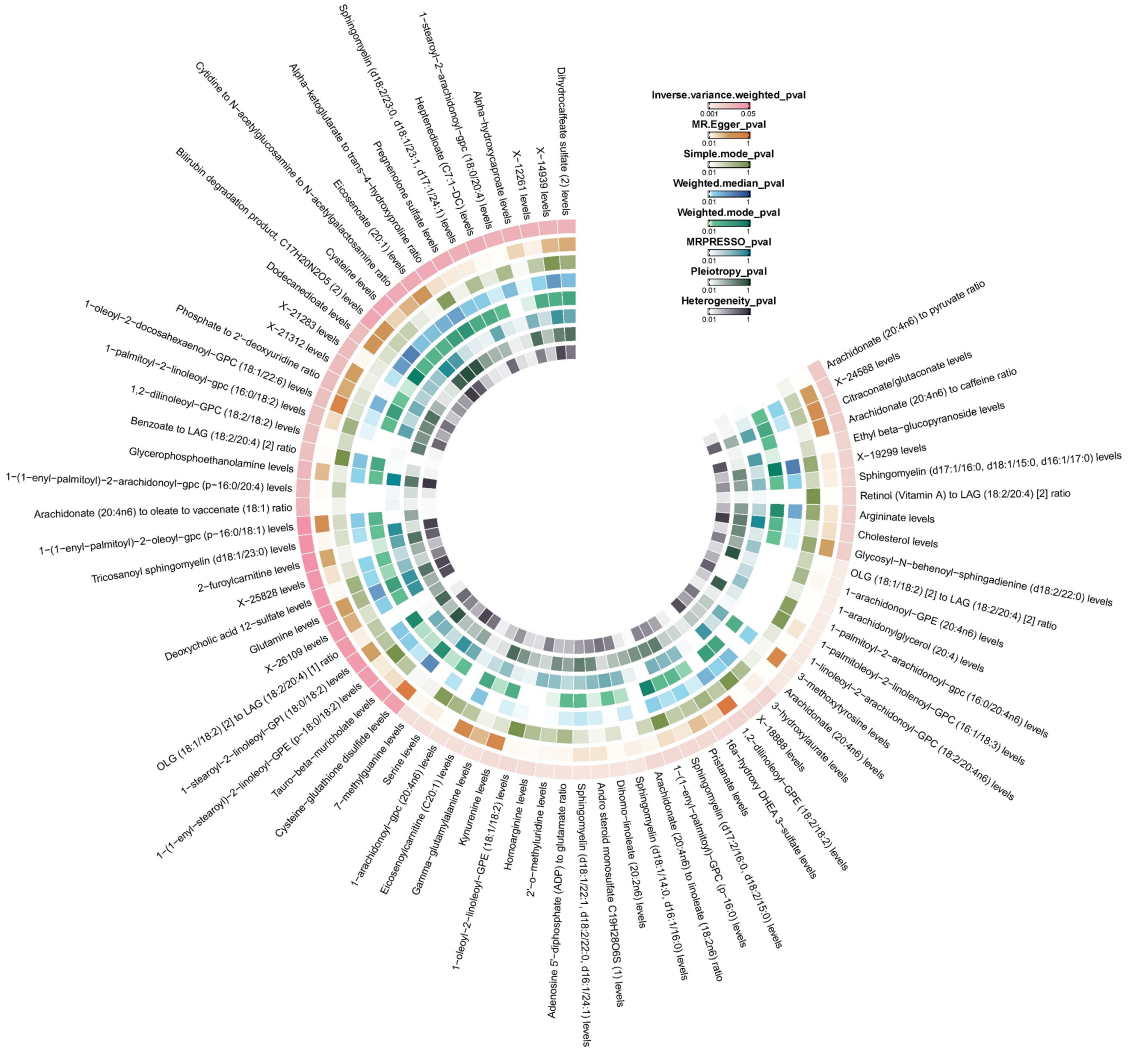
Circle heatmap of the results of five Mendelian randomization analyses and three sensitivity analyses of 77 plasma metabolites on colorectal cancer.

**Figure 3:**
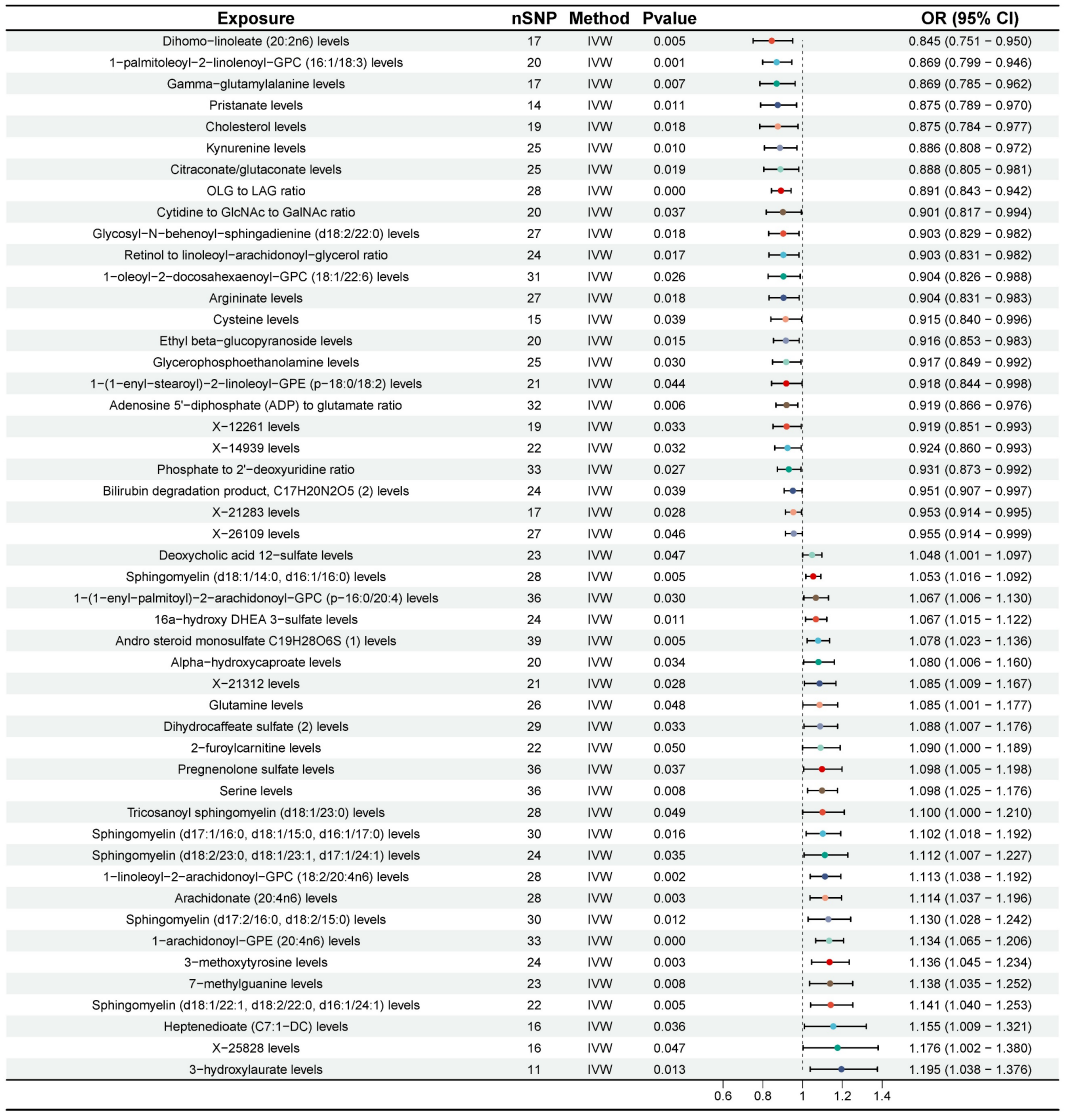
Forest plot for the causality of 49 plasma metabolites on colorectal cancer. CI, confidence interval; IVW, inverse variance weighted; OR, odds ratio; nSNP, number of single nucleotide polymorphisms.

According to the results of IVW method, we initially identified 77 plasma metabolites with potential causal associations with CRC (P < 0.05) (Figure 2). Further selection criteria ultimately identified 49 plasma metabolites with strong causal associations with CRC (Figure 3, Table S2), including 43 reported metabolites and 6 unidentified metabolites. Of the metabolites reported, 23 were associated with an increase in the risk of CRC, including 3-hydroxylaurate, Heptenedioate (C7:1-DC), Sphingomyelin (d18:1/22:1, d18:2/22:0, d16:1/24:1), 7-methylguanine, 1-arachidonoyl-GPE (20:4n6), and other metabolites, with 3-hydroxylauric acid showing the most significant effect (OR = 1.195, 95% CI [1.038, 1.376], P = 0.013). Additionally, 20 metabolites were identified as potential protective factors against CRC, including dihomo-linoleate (20:2n6), 1-palmitoleoyl-2-linolenoyl-GPC (16:1/18:3), Gamma-glutamylalanine, arginine and other metabolites, among which dihomo-linoleate (20:2n6) had the strongest protective effect (OR = 0.845, 95% CI [0.751, 0.950], P = 0.005). Cochran's Q statistics, MR-Egger intercept test, and MR-PRESSO did not indicate any heterogeneity or horizontal pleiotropy. In MR-PRESSO analysis and leave-one-out sensitivity analysis, no single SNP was found to significantly violate the overall effect of plasma metabolites on CRC (Table S2).

### Causal associations of immune cell traits with CRC

**Figure 4:**
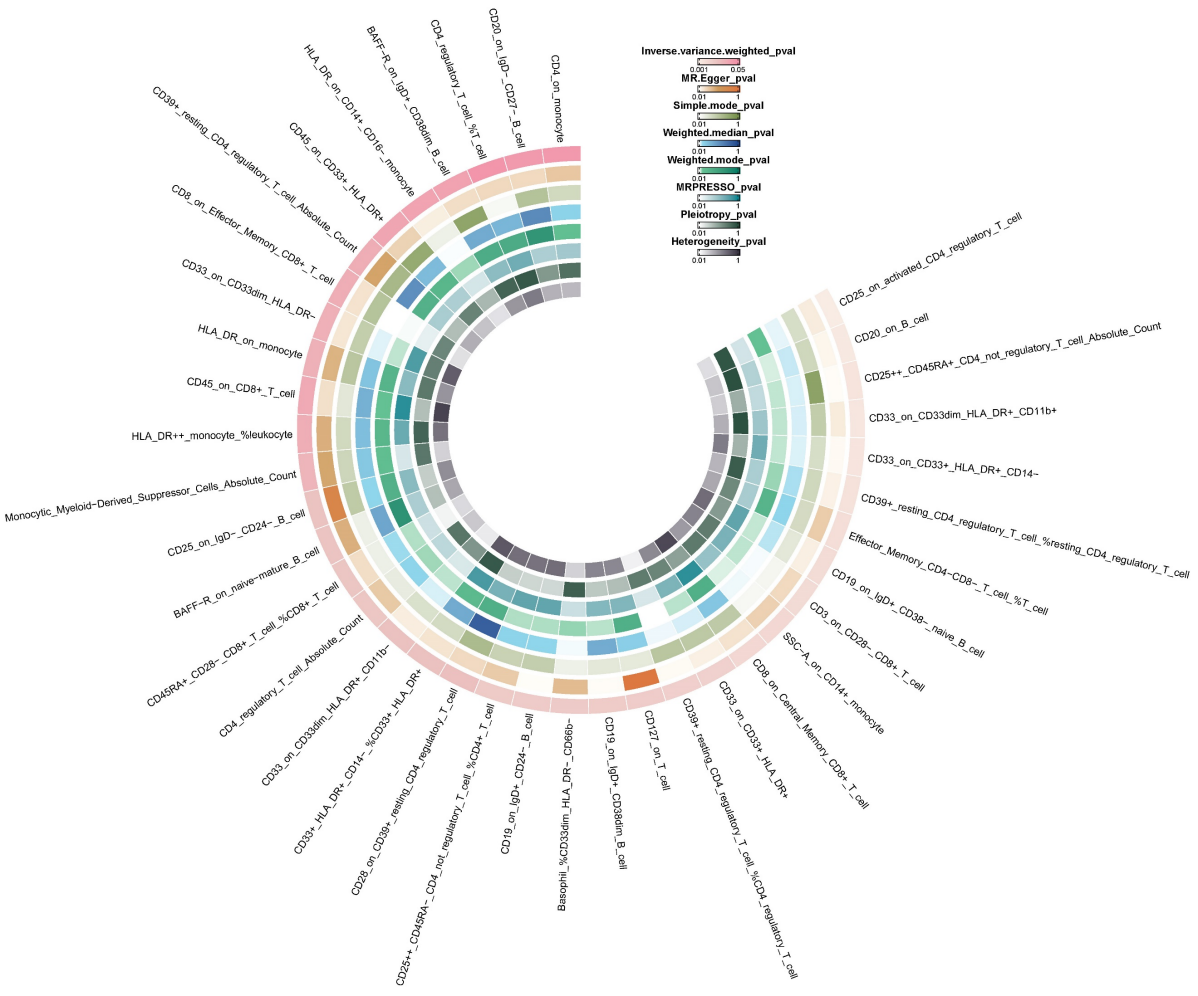
Circle heatmap of the results of five Mendelian randomization analyses and three sensitivity analyses of 38 immune cell traits on colorectal cancer.

**Figure 5:**
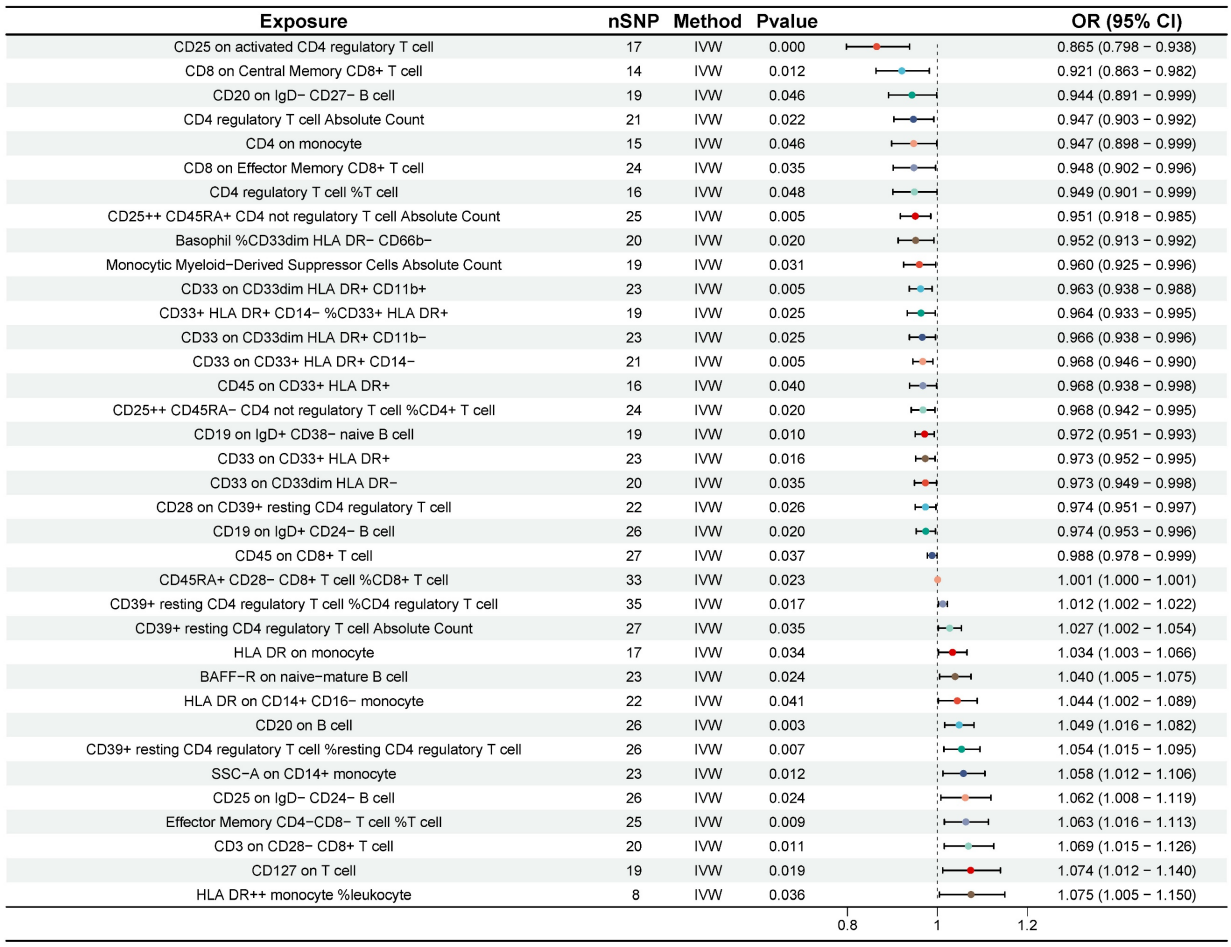
Forest plot for the causality of 36 immune cell traits on colorectal cancer. CI, confidence interval; IVW, inverse variance weighted; OR, odds ratio; nSNP, number of single nucleotide polymorphisms.

Preliminary investigation using the IVW method revealed that 38 immune cell traits were associated with CRC (Figure 4). Further selection criteria ultimately identified 36 immune cell traits with strong causal associations with CRC (Figure 5, Table S3). Among these, 14 immune cell traits were associated with increased risk of CRC, including HLA DR++ monocyte %leukocyte, CD127 on T cell, CD3 on CD28- CD8+ T cell, etc. with HLA DR++ monocyte %leukocyte showing the most significant effect (OR = 1.075, 95% CI [1.005, 1.150], P = 0.036). Additionally, 22 immune cell characteristics were identified as potential protective factors against CRC, such as CD8 on Central Memory CD8+ T cell, CD20 on IgD- CD27- B cell, CD25 on activated CD4 regulatory T cell, and other immune cell traits, with CD25 on activated CD4 regulatory T cell demonstrating the strongest protective effect (OR = 0.865, 95% CI [0.798, 0.938], P < 0.001) (Figure 5). No heterogeneity or horizontal pleiotropy was detected by Cochran's Q statistic, MR-Egger intercept test and MR-PRESSO. In addition, no single SNP was found to significantly violate the overall effect of immune cells on CRC in the MR-PRESSO analysis and in the leave-one-out sensitivity analysis (Table S3).

### Mediating analysis of immune cells traits in the causal association between plasma metabolites and CRC

**Figure 6:**
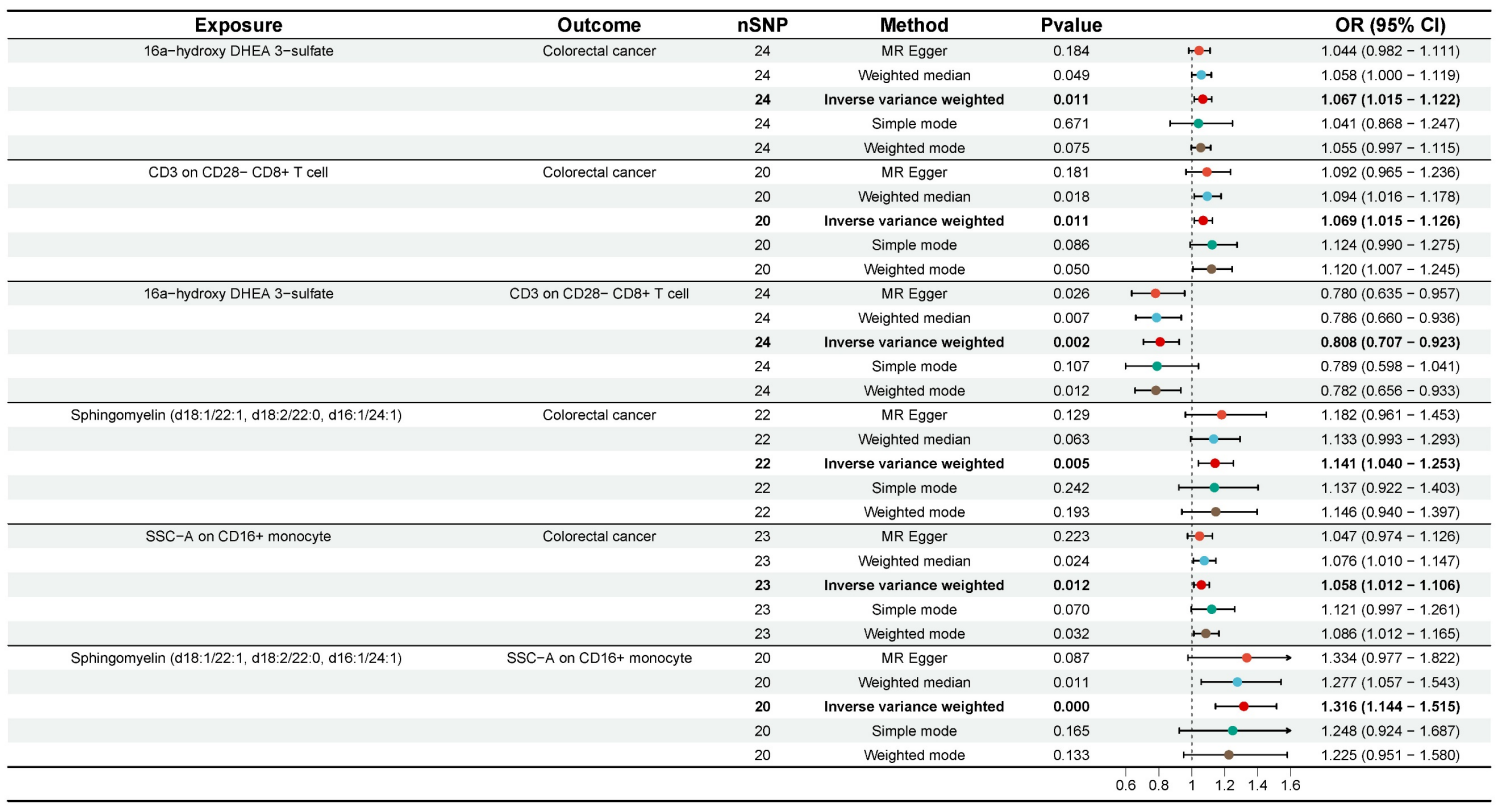
Forest plots of plasma metabolites, immune cell traits, and colorectal cancer that constitute the mediating pathway. CI, confidence interval; OR, odds ratio; nSNP, number of single nucleotide polymorphisms.

Subsequently, we employed a two-step MR analysis to identify immune cells traits that potentially mediate the plasma metabolite-CRC causal effects. The two-sample MR analysis from plasma metabolites to immune cell traits was first performed using 49 plasma metabolites and 36 immune cell traits obtained from the previous analysis. The results showed causal relationships between 30 plasma metabolites and 27 immune cell traits (Table S4). For these plasma metabolites-immune cell traits, effect sizes (beta A) were calculated. In the second step, the 27 immune cells traits identified in the first step were used as exposures. After adjusting for the effects of the plasma metabolites, we again performed two-sample MR analyses of immune cells traits-CRC and calculated their effector values (beta B). To date, 51 plasma metabolites-immune cell traits-CRC pathways have been tentatively identified. Subsequently, one mediation pathway was removed due to the presence of a reverse causal association. Finally, the coefficient product method was used to calculate mediated effects, mediated proportion, and P value for each identified mediation pathway (Table S5). Ultimately, we identified two significant mediation pathways (P < 0.05). First, CD3 on CD28- CD8+ T cell mediated the pathway from 16a-hydroxy DHEA 3-sulfate to CRC with a mediation effect of -0.014 (95% CI [-0.043, 0.014], P = 0.048, Table 1, Figure 6), accounting for -22.06% of the total effect. Second, SSC-A on CD14+ monocyte mediated the pathway from Sphingomyelin (d18:1/22:1, d18:2/22:0, d16:1/24:1) to CRC with a mediation effect of 0.0155 (95% CI [-0.023, 0.054], P = 0.036), accounting for 11.72% of the total effect (Table 1, Figure 6). These results effectively elucidate the causal relationships between plasma metabolites and CRC mediated by immune cell traits.

### Infiltration of CD28- CD8+ T cells and CD14+ monocytes in CRC and normal colorectal tissues

**Figure 7:**
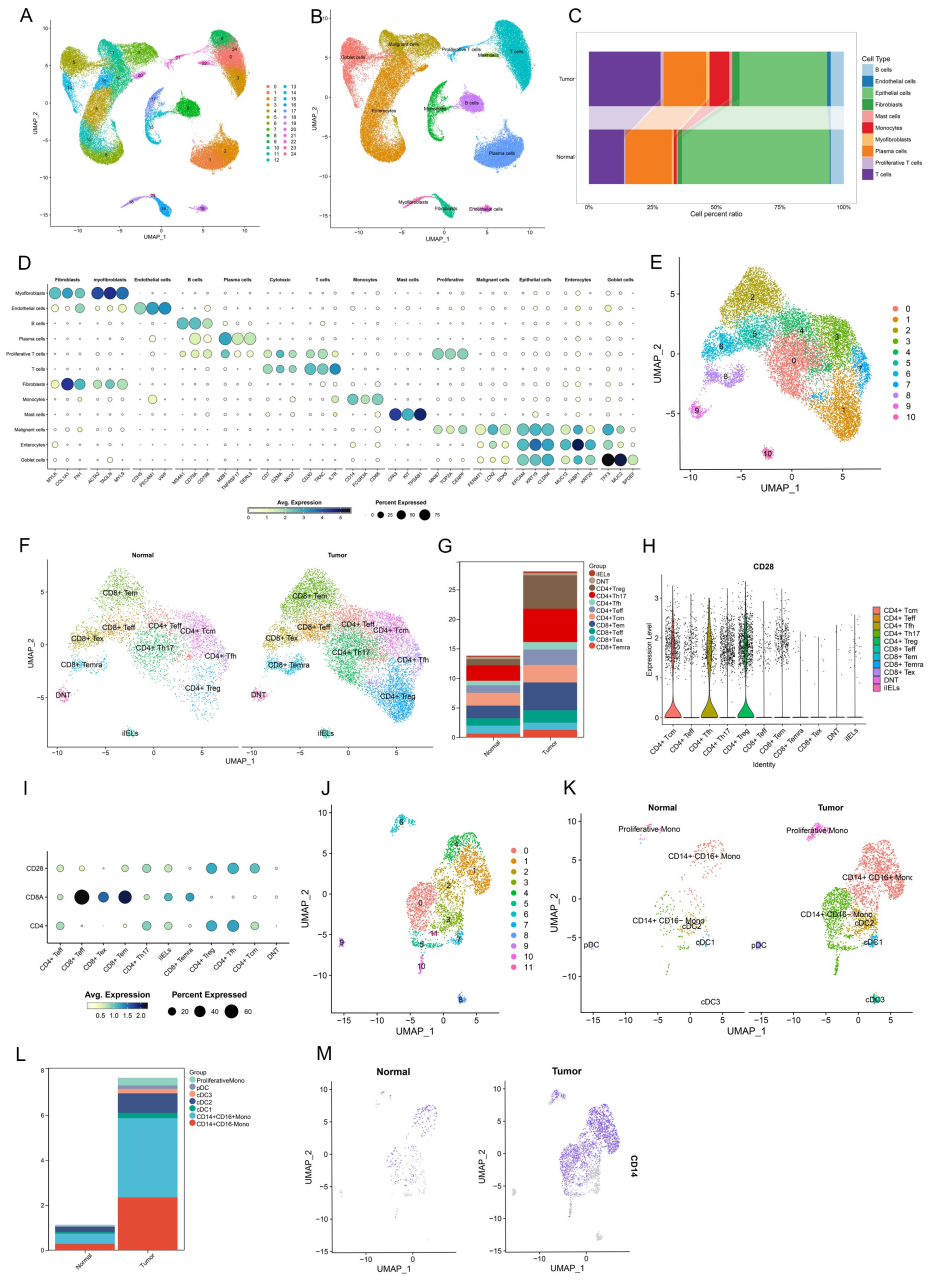
Single-cell transcriptional landscape of diverse cell types in CRC and adjacent normal tissue microenvironments. (A, B) Clusters representing primary immune cell types observed in CRC and the tumor microenvironment of neighboring normal tissues. (C) A stacked bar chart depicts the relative proportions of major cell types in CRC and adjacent normal samples. (D) A bubble chart highlights the molecular markers associated with 11 principal cell types. (E, F) UMAP visualizations of 11 T cell subpopulations. (G) A stacked bar chart illustrates the distribution of different T cell subpopulations in CRC compared to adjacent normal samples. (H) A violin plot demonstrating the relative expression levels of CD28 among T cell subpopulations. (I) A bubble chart depicting the relative expression of CD28, CD8, and CD4 within T cell subpopulations. (J, K) UMAP representations of 7 distinct monocyte subpopulations. (L) A stacked bar chart presenting the distribution of various monocyte subpopulations in CRC and adjacent normal samples. (M) UMAP projection showing the expression and spatial distribution of CD14 in monocytes, with each point representing a cell characterized by positive marker gene expression.

To investigate the infiltration of CD28-CD8+ T cells and CD14+ monocytes in CRC and normal colorectal tissues, scRNA-seq data from 12 patients (12 CRC tissues and 12 paired normal tissues) were analyzed. After filtering out low-quality cells based on selection criteria, 65,878 cells were included in the analysis. Using the FindVariableFeatures function, the top 2,000 highly variable genes were selected for further analysis. Dimensionality reduction, clustering, and annotation identified 12 cell types, including T cells (CD3D, TRAC, IL7R), monocytes (CD14, FCGR3A, CD68), fibroblasts (MYLK, COL1A1, FN1), myofibroblasts (ACAT2, TAGLN, MYL9), endothelial cells (CDH5, PECAM1, VWF), B cells (MS4A1, CD79A, CD79B), plasma cells (MZB1, TNFRSF17, DERL3), mast cells (CPA3, KIT, TPSAB1), malignant cells (FERMT1, LCN2, SOX9), intestinal epithelial cells (MUC12, FABP1, KRT20), and goblet cells (TFF3, MUC2, SPDEF) (Figure 7A, B, D). Further analysis revealed that monocytes and T cells proportions were significantly elevated in CRC compared to normal tissues (T cells: 28.085% vs 13.794%; monocytes: 7.666% vs 1.128%.) (Figure 7C, Table S6). Upon additional dimensionality reduction, clustering, and annotation, T cells were categorized into 11 subpopulations, including four CD8+ T cell subtypes: effector CD8+ T cells (CD8+ Teff), effector memory CD8+ T cells (CD8+ Tem), terminally differentiated effector memory or effector CD8+ T cells (CD8+ Temra), and exhausted CD8+ T cells (CD8+ Tex) (Figure 7E, F). Monocytes were divided into seven subpopulations, including CD14+ CD16- monocytes, CD14+ CD16+ monocytes, proliferative monocytes, conventional dendritic cells (cDC1, cDC2, cDC3), and plasmacytoid dendritic cells (pDC) (Figure 7J, K). Further analysis revealed that CD28- CD8+ T cells were primarily composed of CD8+ Temra and CD8+ Tex cells, with a higher infiltration rate in CRC compared to normal tissues (2.464% vs 1.969%) (Figure 7G, Table S7). Similarly, CD14+ monocytes exhibited a higher infiltration rate in CRC compared to normal tissues (5.875% vs 0.731%) (Figure 7L, Table S8). These findings suggest that both CD28- CD8+ T cells and CD14+ monocytes show increased infiltration in CRC, indicating their potential involvement in CRC development. These results align with our MR analysis, further supporting the causal role of these two subpopulations in CRC risk.

### Metabolic activity of CD28- CD8+ T cells and CD14+ monocytes and their interaction with malignant cells

**Figure 8:**
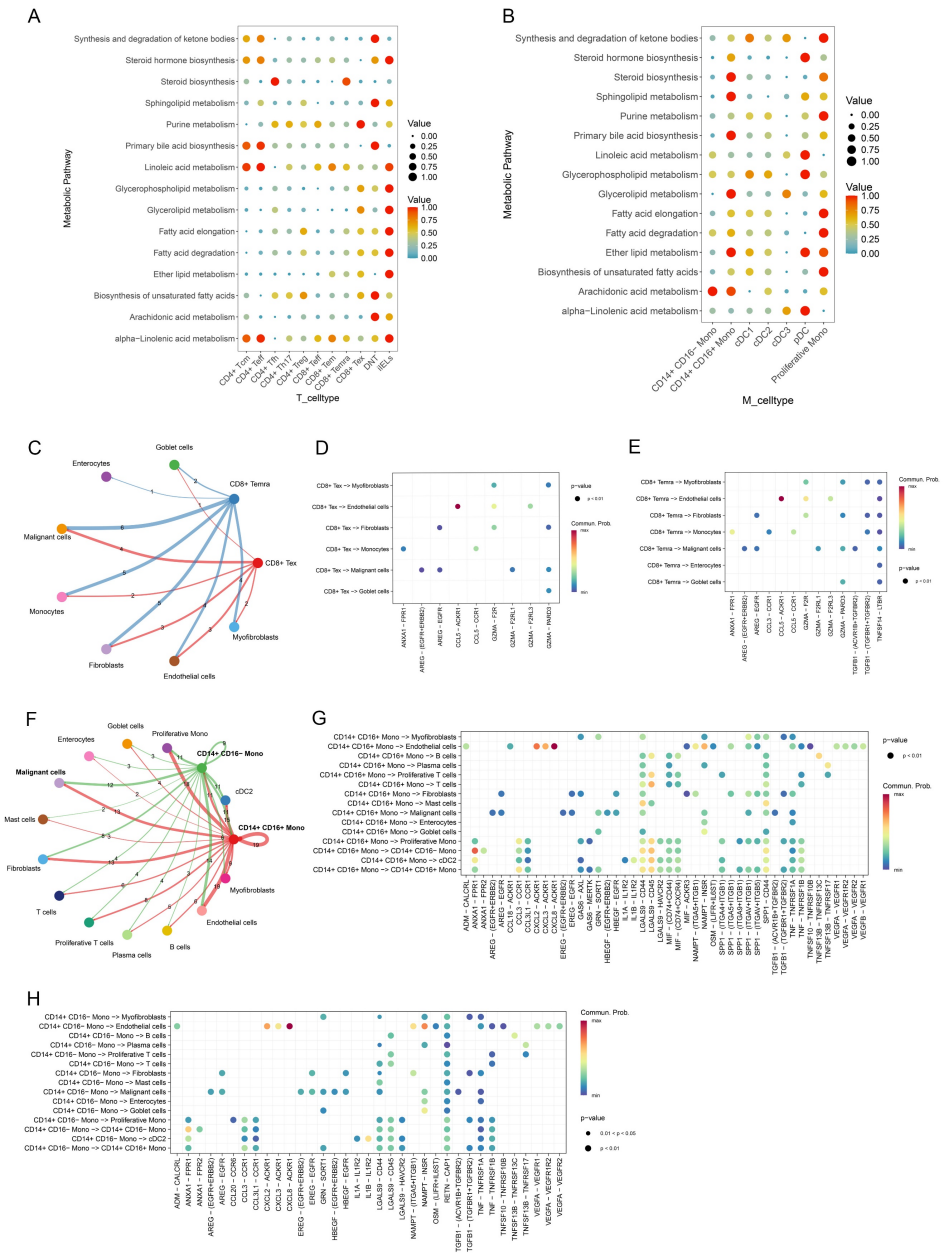
Metabolic alterations of CD28- CD8+ T cells and CD14+ monocytes in CRC and their interactions with malignant cells. (A, B) Bubble plots illustrating the enrichment of metabolic pathways in T-cell subsets (A) and monocyte subsets (B) as calculated by scMetabolism. (C) Interactions between CD28- CD8+ T cells (including CD8+ Temra and CD8+ Tex cells) and other cell types, with line thickness representing the number of ligand-receptor pairs mediating communication. (D) Communication probabilities of significant ligand-receptor pairs from CD8+ Tex cells to various cell types in CRC, with point colors reflecting communication probabilities; absence of color indicates zero communication probability. (E) Communication probabilities of significant ligand-receptor pairs from CD8+ Temra cells to various cell types in CRC. (F) Interactions between CD14+ monocytes (comprising CD14+ CD16- monocytes and CD14+ CD16+ monocytes) and other cell types, with line thickness indicating the number of receptor-ligand pairs involved in communication. (G) Communication probabilities for significant pairs from CD14+ CD16+ monocytes to other cell types in CRC. (H) Communication probabilities for pairs from CD14+ CD16- monocytes to various cell types.

To explore the metabolic states of CD28- CD8+ T cells and CD14+ monocytes, the "scMetabolism" package was used to score active metabolic pathways. Results showed a significant upregulation of the steroid biosynthesis pathway in CD8+ Temra cells, and sphingolipid metabolism was notably enriched in CD14+ CD16+ monocytes (Figure 8A, B). Additionally, cell communication analysis revealed the interactions between CD28- CD8+ T cells (CD8+ Temra, CD8+ Tex) and CD14+ monocytes with other cells in the CRC microenvironment (Figure 8C, F). Specifically, CD8+ Temra and CD8+ Tex cells were found to interact with malignant cells via the GZMA-PARD3 (Figure 8D, E). CD14+ CD16+ monocytes communicated with malignant cells through LGALS9-CD44 and SPP1-CD44, etc. (Figure 8G), while NAMPT-INSR signaling in CD14+ CD16- monocytes was the most prominent in interactions with malignant cells (Figure 8H).

## Discussion

In the past decade, with advancements in metabolomics technologies, numerous studies have increasingly recognized the pivotal role of plasma metabolites in oncology. Specific plasma metabolites have shown promising predictive value in the diagnosis and prognosis of tumors 23, 24. Variations in the types and levels of plasma metabolites have been observed among CRC patients at different stages 25. To investigate the causal relationship between plasma metabolites and CRC, we conducted a two-sample MR study and identified 49 plasma metabolites causally associated with CRC, consistent with previous research findings 26. Sphingomyelins, crucial lipid molecules, play key roles in cellular membrane structure and function, as well as biological functions such as cell signaling, membrane fluidity, and specific cell-cell recognition 27. Studies have indicated significant increases in sphingomyelins (d18:1/16:0) in exosomes derived from primary CRC cells 28. The molecules independently contribute to CRC risk and possess diagnostic significance 26, 29. Furthermore, 16a-hydroxy-DHEA-3-sulfate has been identified as an independent risk factor for invasive breast cancer 30. Our study results demonstrate a causal relationship between sphingomyelins, 16a-hydroxy-DHEA-3-sulfate, and CRC, where they act as independent risk factors for CRC. Additionally, a study utilizing MR analysis found that an increase in circulating eosinophil counts may have a potential protective effect against CRC 17. However, our two-sample MR analysis identified 36 immune cell traits causally linked to CRC, of which 14 traits were found to have adverse effects and 22 traits were associated with protective effects. This research provides a broader analysis of immune cells and their traits, offering new insights within the existing knowledge framework.

Metabolites involved in the regulation of tumor immunity have also been identified in recent studies 31-33. To investigate whether plasma metabolites affect CRC by modulating immune cells, we further conducted a two-step MR analysis. We found that two plasma metabolites, Sphingomyelin (d18:1/22:1, d18:2/22:0, d16:1/24:1) and 16a-hydroxy-DHEA-3-sulfate, influence CRC through mediation by two immune cell traits, SSC-A on CD14+ monocyte and CD3 on CD28- CD8+ T cell. CD14+ monocytes are essential in the immune system, particularly in the innate immune reaction to pathogens and tissue damage 34. They are implicated in the pathogenesis of various diseases including inflammation, atherosclerosis, tumors, and metabolic disorders 35. In the tumor microenvironment, peripheral blood CD14+ monocytes can differentiate into macrophages or DCs, contributing to the formation of an immunosuppressive environment that promotes tumor progression 35, 36. In our study, scRNA-seq analysis confirmed that the proportion of CD14+ monocytes in CRC tissue is higher than that in normal colon tissue. Studies have also indicated that soluble CD14 secreted by monocytes supports the survival of chronic lymphocytic leukemia cells 37. SSC-A (side scatter - area) is a parameter used in flow cytometry to describe cellular morphological features such as size, complexity, and granularity, which can distinguish different cell subsets and assess cell states. Using parameters such as SSC-A, changes in the activation and function of CD14+ monocytes can be detected. Currently, there have been no reports investigating whether CD14+ monocytes mediate the effects of plasma metabolites on the occurrence and progression of CRC. Therefore, our study identifies a causal relationship between SSC-A on CD14+ monocyte and CRC, serving as a mediator for the impact of Sphingomyelin (d18:1/22:1, d18:2/22:0, d16:1/24:1) on CRC. Additionally, scRNA-seq confirmed that elevated sphingolipid metabolism levels occur in CD14+ monocytes, which interact with tumor cells. This finding is consistent with our MR results, suggesting that CD14+ monocytes, influenced by sphingolipid metabolism, may play a role in the onset and progression of CRC.

In addition, we identified a causal relationship between CD3 on CD28- CD8+ T cell and CRC. CD3 and CD28 are two crucial costimulatory molecules on the surface of T cells. CD3 is part of the T cell receptor complex, involved in T cell signaling, while CD28 acts as a costimulatory molecule, binding to B7 molecules (such as CD80 and CD86) on antigen-presenting cells (e.g., DCs), promoting T cell activation and proliferation. CD3+ CD28- CD8+ T cells represent a subset of CD8+ T cells characterized by the absence of the costimulatory molecule CD28. These cells are typically found in the tumor microenvironment and are associated with T cell dysfunction and senescence 38. In our study, scRNA-seq analysis revealed that CD28- CD8+ T cells are predominantly composed of exhausted CD8+ T cells and CD8+ Temra. Furthermore, the proportion of these cells in CRC tumor tissue was found to be higher than that in normal colonic tissue. Elevated peripheral blood CD28- CD8+ T cells have been linked to poorer prognosis in metastatic breast cancer 39. Lung squamous cell carcinoma patients with more CD8+ CD28-T cells have worse prognosis than those with fewer 40. CD8+ CD28- T regulatory lymphocytes can inhibit T cell proliferation and cytotoxicity, potentially facilitating immune evasion by tumors 41. Chronic antigen stimulation found in cancer, leads to the accumulation of CD28- CD8+ T cells, further promoting tumor progression 42. Our study not only demonstrates the relationship between CD3 on CD28- CD8+ T cell and CRC but also suggests its role as a crucial modulator in the causal association of 16a-hydroxy-DHEA-3-sulfate with CRC. 16α-hydroxy-DHEA-3-sulfate is a steroid metabolite involved in steroid synthesis. Our scRNA-seq analysis further confirmed a significant enrichment of steroid synthesis pathways in CD28- CD8+ T cells. Additionally, interactions were identified between CRC cells and CD28- CD8+ T cells, indicating that CD28- CD8+ T cells play a mediating role in the effects of 16α-hydroxy-DHEA-3-sulfate plasma metabolites on CRC.

In this study, we investigated the causal role of plasma metabolites on CRC and the mediating role of immune cells in the association of plasma metabolites with CRC by mediated MR analysis combined with scRNA-seq analysis. However, several limitations should be acknowledged. Firstly, we need to realize that there is still potential heterogeneity and horizontal pleiotropy that may not be effectively assessed, although rigorous sensitivity tests have been performed. Secondly, the applicability of the conclusions is limited as all data were derived from European populations, and validation in other populations is necessary. Thirdly, two plasma metabolites exerted effects on CRC through two immune cell traits, with relatively small mediation percentages, suggesting potential involvement of other mediators warranting further investigation. Fourthly, this study validated the results of MR exclusively through scRNA-seq analysis, and further experimental validation is needed to elucidate the specific regulatory mechanisms.

## Conclusion

The study indicated a causal association between plasma metabolites, immune cell traits and elevated CRC risk. SSC-A on CD14+ monocyte, CD3 on CD28- CD8+ T cell mediated the effects of Sphingomyelin (d18:1/22:1, d18:2/22:0, d16:1/ 24:1), and 16a-hydroxy-DHEA-3-sulfate on CRC risk. These findings elucidate potential mechanisms between plasma metabolites, immune cell traits, and CRC.

## Supplementary Material

Supplementary tables.

## Figures and Tables

**Table 1:** Mediation analysis of the effect of plasma metabolites on colorectal cancer via immune cell traits

Mediation Pathway	Mediated effect β (95% CI)	Mediated proportion (%)	P value	Z value
Sphingomyelin (d18:1/22:1, d18:2/22:0, d16:1/24:1)/SSC-A on CD14+ monocyte/Colorectal cancer	0.016 (-0.023, 0.054)	11.723	0.036	2.096
16a-hydroxy DHEA 3-sulfate/CD3 on CD28- CD8+ T cell/Colorectal cancer	-0.014 (-0.043, 0.014)	-22.064	0.048	-1.977

## Abbreviations

CRC: colorectal cancer
GWAS: genome-wide association studies
MR: Mendelian randomization
DCs: dendritic cells
AC: absolute cell counts
RC: relative cell counts
MFI: median fluorescence intensity
MP: morphological parameters
IV: instrumental variable
SNP: single nucleotide polymorphism
MAF: minor allele frequency
IVW: Inverse Variance Weighted
OR: Odds ratio
CI: confidence interval
scRNA-seq: single-cell RNA sequencing
GEO: Gene Expression Omnibus
UMAP: Uniform Manifold Approximation and Projection
CD8+ Teff: effector CD8+ T cells
CD8+ Tem: effector memory CD8+ T cells
CD8+ Temra: terminally differentiated effector memory or effector CD8+ T cells
CD8+ Tex: exhausted CD8+ T cells
cDC: conventional dendritic cells
pDC: plasmacytoid dendritic cells

## References

[1] Sung H, Ferlay J, Siegel RL, Laversanne M, Soerjomataram I, Jemal A (2021). Global Cancer Statistics 2020: GLOBOCAN Estimates of Incidence and Mortality Worldwide for 36 Cancers in 185 Countries. CA Cancer J Clin.

[2] Tauriello DV, Calon A, Lonardo E, Batlle E (2017). Determinants of metastatic competency in colorectal cancer. Mol Oncol.

[3] Bull CJ, Bell JA, Murphy N, Sanderson E, Davey Smith G, Timpson NJ (2020). Adiposity, metabolites, and colorectal cancer risk: Mendelian randomization study. BMC Med.

[4] Bardou M, Barkun AN, Martel M (2013). Obesity and colorectal cancer. Gut.

[5] Martin-Perez M, Urdiroz-Urricelqui U, Bigas C, Benitah SA (2022). The role of lipids in cancer progression and metastasis. Cell Metab.

[6] Bai Z, Zhou Y, Ye Z, Xiong J, Lan H, Wang F (2021). Tumor-Infiltrating Lymphocytes in Colorectal Cancer: The Fundamental Indication and Application on Immunotherapy. Front Immunol.

[7] Chen Y, Lu T, Pettersson-Kymmer U, Stewart ID, Butler-Laporte G, Nakanishi T (2023). Genomic atlas of the plasma metabolome prioritizes metabolites implicated in human diseases. Nat Genet.

[8] Hanahan D, Weinberg RA (2011). Hallmarks of cancer: the next generation. Cell.

[9] Wolrab D, Jirásko R, Cífková E, Höring M, Mei D, Chocholoušková M (2022). Lipidomic profiling of human serum enables detection of pancreatic cancer. Nat Commun.

[10] Lu Y, Li D, Wang L, Zhang H, Jiang F, Zhang R (2023). Comprehensive Investigation on Associations between Dietary Intake and Blood Levels of Fatty Acids and Colorectal Cancer Risk. Nutrients.

[11] Jennemann R, Volz M, Bestvater F, Schmidt C, Richter K, Kaden S (2021). Blockade of Glycosphingolipid Synthesis Inhibits Cell Cycle and Spheroid Growth of Colon Cancer Cells In Vitro and Experimental Colon Cancer Incidence In Vivo. Int J Mol Sci.

[12] Li S, Yang H, Li W, Liu JY, Ren LW, Yang YH (2022). ADH1C inhibits progression of colorectal cancer through the ADH1C/PHGDH /PSAT1/serine metabolic pathway. Acta Pharmacol Sin.

[13] Mihajlović M, Ninić A, Ostojić M, Sopić M, Stefanović A, Vekić J (2022). Association of Adiponectin Receptors with Metabolic and Immune Homeostasis Parameters in Colorectal Cancer: In Silico Analysis and Observational Findings. Int J Environ Res Public Health.

[14] Chen Y, Xie Y, Ci H, Cheng Z, Kuang Y, Li S (2024). Plasma metabolites and risk of seven cancers: a two-sample Mendelian randomization study among European descendants. BMC Med.

[15] Cong J, Liu P, Han Z, Ying W, Li C, Yang Y (2024). Bile acids modified by the intestinal microbiota promote colorectal cancer growth by suppressing CD8(+) T cell effector functions. Immunity.

[16] Tallima H, Azzazy HME, El Ridi R (2021). Cell surface sphingomyelin: key role in cancer initiation, progression, and immune evasion. Lipids Health Dis.

[17] Constantinescu AE, Bull CJ, Jones N, Mitchell R, Burrows K, Dimou N (2024). Circulating white blood cell traits and colorectal cancer risk: A Mendelian randomisation study. Int J Cancer.

[18] Hay ZLZ, Slansky JE (2022). Granzymes: The Molecular Executors of Immune-Mediated Cytotoxicity. Int J Mol Sci.

[19] Deng Y, Hu JC, He SH, Lou B, Ding TB, Yang JT (2021). Sphingomyelin synthase 2 facilitates M2-like macrophage polarization and tumor progression in a mouse model of triple-negative breast cancer. Acta Pharmacol Sin.

[20] Morris JA, Sun JS, Sanjana NE (2024). Next-generation forward genetic screens: uniting high-throughput perturbations with single-cell analysis. Trends Genet.

[21] Kurki MI, Karjalainen J, Palta P, Sipilä TP, Kristiansson K, Donner KM (2023). FinnGen provides genetic insights from a well-phenotyped isolated population. Nature.

[22] Orrù V, Steri M, Sidore C, Marongiu M, Serra V, Olla S (2020). Complex genetic signatures in immune cells underlie autoimmunity and inform therapy. Nat Genet.

[23] Wang Z, Cui B, Zhang F, Yang Y, Shen X, Li Z (2019). Development of a Correlative Strategy To Discover Colorectal Tumor Tissue Derived Metabolite Biomarkers in Plasma Using Untargeted Metabolomics. Anal Chem.

[24] Huang S, Guo Y, Li ZW, Shui G, Tian H, Li BW (2021). Identification and Validation of Plasma Metabolomic Signatures in Precancerous Gastric Lesions That Progress to Cancer. JAMA Netw Open.

[25] Geijsen A, van Roekel EH, van Duijnhoven FJB, Achaintre D, Bachleitner-Hofmann T, Baierl A (2020). Plasma metabolites associated with colorectal cancer stage: Findings from an international consortium. Int J Cancer.

[26] Sun J, Zhao J, Zhou S, Li X, Li T, Wang L (2024). Systematic investigation of genetically determined plasma and urinary metabolites to discover potential interventional targets for colorectal cancer. J Natl Cancer Inst.

[27] Slotte JP (2013). Biological functions of sphingomyelins. Prog Lipid Res.

[28] Elmallah MIY, Ortega-Deballon P, Hermite L, Pais-De-Barros JP, Gobbo J, Garrido C (2022). Lipidomic profiling of exosomes from colorectal cancer cells and patients reveals potential biomarkers. Mol Oncol.

[29] Chen H, Zhou H, Liang Y, Huang Z, Yang S, Wang X (2023). UHPLC-HRMS-based serum untargeted lipidomics: Phosphatidylcholines and sphingomyelins are the main disturbed lipid markers to distinguish colorectal advanced adenoma from cancer. J Pharm Biomed Anal.

[30] Moore SC, Playdon MC, Sampson JN, Hoover RN, Trabert B, Matthews CE (2018). A Metabolomics Analysis of Body Mass Index and Postmenopausal Breast Cancer Risk. J Natl Cancer Inst.

[31] King RJ, Singh PK, Mehla K (2022). The cholesterol pathway: impact on immunity and cancer. Trends Immunol.

[32] Kazak L, Cohen P (2020). Creatine metabolism: energy homeostasis, immunity and cancer biology. Nat Rev Endocrinol.

[33] Jia D, Wang Q, Qi Y, Jiang Y, He J, Lin Y (2024). Microbial metabolite enhances immunotherapy efficacy by modulating T cell stemness in pan-cancer. Cell.

[34] Sharygin D, Koniaris LG, Wells C, Zimmers TA, Hamidi T (2023). Role of CD14 in human disease. Immunology.

[35] Wu Z, Zhang Z, Lei Z, Lei P (2019). CD14: Biology and role in the pathogenesis of disease. Cytokine Growth Factor Rev.

[36] Zarif JC, Hernandez JR, Verdone JE, Campbell SP, Drake CG, Pienta KJ (2016). A phased strategy to differentiate human CD14+monocytes into classically and alternatively activated macrophages and dendritic cells. Biotechniques.

[37] Seiffert M, Schulz A, Ohl S, Döhner H, Stilgenbauer S, Lichter P (2010). Soluble CD14 is a novel monocyte-derived survival factor for chronic lymphocytic leukemia cells, which is induced by CLL cells in vitro and present at abnormally high levels in vivo. Blood.

[38] Huff WX, Kwon JH, Henriquez M, Fetcko K, Dey M (2019). The Evolving Role of CD8(+)CD28(-) Immunosenescent T Cells in Cancer Immunology. Int J Mol Sci.

[39] Song Q, Ren J, Zhou X, Wang X, Song G, Hobeika A (2018). Circulating CD8(+)CD28(-) suppressor T cells tied to poorer prognosis among metastatic breast cancer patients receiving adoptive T-cell therapy: A cohort study. Cytotherapy.

[40] Liu C, Jing W, An N, Li A, Yan W, Zhu H (2019). Prognostic significance of peripheral CD8+CD28+ and CD8+CD28- T cells in advanced non-small cell lung cancer patients treated with chemo(radio)therapy. J Transl Med.

[41] Filaci G, Fenoglio D, Fravega M, Ansaldo G, Borgonovo G, Traverso P (2007). CD8+ CD28- T regulatory lymphocytes inhibiting T cell proliferative and cytotoxic functions infiltrate human cancers. J Immunol.

[42] Strioga M, Pasukoniene V, Characiejus D (2011). CD8+ CD28- and CD8+ CD57+ T cells and their role in health and disease. Immunology.

